# Identification of claudin-4 binder that attenuates tight junction barrier function by TR-FRET-based screening assay

**DOI:** 10.1038/s41598-017-15108-y

**Published:** 2017-11-06

**Authors:** Akihiro Watari, Miki Kodaka, Koji Matsuhisa, Yuta Sakamoto, Kota Hisaie, Norihito Kawashita, Tatsuya Takagi, Yoshiaki Yamagishi, Hidehiko Suzuki, Hirofumi Tsujino, Kiyohito Yagi, Masuo Kondoh

**Affiliations:** 1 Graduate School of Pharmaceutical Sciences, Osaka University, 1-6 Yamadaoka, Suita, Osaka, 565-0871 Japan; 2 Department of Stress Protein Processing, Institute of Biomedical and Health Sciences, Hiroshima University, Hiroshima, Japan; 3 Faculty of Science and Engineering, Kindai University 3-4-1 Kowakae, Higashiosaka City, Osaka, 577-8502 Japan; 4 Research Institute for Microbial Diseases, Osaka University, 3-1 Yamadaoka, Suita, Osaka, 565-0871 Japan; 5 Research Institute of Pharmaceutical Sciences, Musashino University, 1-1-20 Shinmachi, Nishi-Tokyo, 202-8585, Japan; 6 Laboratory of Vaccine Materials and Laboratory of Gut Environmental System, National Institutes of Biomedical Innovation, Health and Nutrition (NIBIOHN), Osaka, 567-0085 Japan

## Abstract

Claudins are key functional and structural components of tight junctions (TJs) in epithelial cell sheets. The C-terminal fragment of *Clostridium perfringens* enterotoxin (C-CPE) binds to claudin-4 and reversibly modulates intestinal TJ seals, thereby enhancing paracellular transport of solutes. However, the use of C-CPE as an absorption enhancer is limited by the molecule’s immunogenicity and manufacturing cost. Here, we developed a high-throughput screening system based on the Time-Resolved Fluorescence Resonance Energy Transfer (TR-FRET) method to identify claudin-4 binders in a library collection of 32,560 compounds. Thiostrepton, identified from the screen, decreased transepithelial electrical resistance and increased flux of 4-kDa fluorescein isothiocyanate–labelled dextran (FD-4) in Caco-2 cell monolayers, a model of intestinal epithelium. Thiostrepton changed the expression, but not the localisation, of TJ components. Treatment of rat jejunum with thiostrepton increased the absorption of FD-4 without tissue toxicity, indicating that thiostrepton is a novel claudin-4 binder that enhances intestinal permeability. The screening system may therefore be a useful tool for identifying claudin-4 binders to enhance drug absorption in mucosa.

## Introduction

Biopharmaceuticals, including peptides, proteins, antibodies and nucleic acids, play pivotal roles in the development of new drugs with breakthrough effects. The proportion of the drug market occupied by biopharmaceuticals is increasing yearly. Currently, biopharmaceuticals are administered primarily by injection, despite the pain and potential adverse effects (e.g., anaphylaxis) associated with this route. Therefore, the development of biopharmaceuticals that can be given through oral, nasal, pulmonary or percutaneous administration is required to avoid the risks of injection. However, biopharmaceuticals show remarkably low rates of absorption through mucosal or cutaneous epithelium owing to their hydrophilicity and high molecular weight^1^, and we need to develop a method for increasing their absorption to ensure sufficient bioavailability^2^.

Tight junctions (TJs), which are formed in the interstices between adjacent epithelial cells, seal the paracellular space to restrict the passage of solutes through the epithelial cell sheet^3,4^. Because TJs limit drug permeation through paracellular spaces, regulating TJ seals is an efficacious strategy for improving drug absorption^5^. A variety of substances controlling paracellular permeability have been developed^5–12^, and several of these bind claudin, a major component of the TJ barrier^13,14^. One of the most efficient is the C-terminal fragment of *Clostridium perfringens* enterotoxin (C-CPE), which binds to claudin-4 and can reversibly weaken the TJ barrier^15^. In rats, C-CPE increases the jejunal absorption of 4-kDa dextran (FD-4) by 400-fold over that of the currently used clinical absorption enhancer, sodium caprate^8^. Furthermore, C-CPE promotes the absorption of human parathyroid hormone, not only via the intestine, but also via the nasal and pulmonary routes^16^. Therefore, modulation of claudin-4 is a promising strategy for enhancing drug absorption by the paracellular permeation route. However, C-CPE is derived from a toxin fragment, and its immunogenicity may increase with multiple administrations^17^. In addition, it is desirable to develop a low-molecular–weight claudin binder that can be mass-produced at a low cost.

Here, we constructed a high-throughput screening system based on the Time-Resolved Fluorescence Resonance Energy Transfer (TR-FRET) method for identifying claudin-4 binders to regulate mucosal TJ-barrier function^18^. We then used a TR-FRET–based assay to screen 32,560 compounds. The screen identified thiostrepton as a novel claudin-4 binder that attenuates TJ-barrier function *in vitro* and *in vivo*. We expect that the TR-FRET–based screening assay will be useful for identifying compounds that modulate the permeability of the mucosal epithelial barrier.

## Results

### Preparation of detection assay for claudin-4 and C-CPE interaction by TR-FRET

C-CPE is highly active in lowering TJ barrier function, suggesting that (a) the region of interaction between C-CPE and claudin-4 is pivotal in the disruption of claudin-4-mediated TJ barrier formation; and (b) interactions between other claudin-4 binders and this region have the potential to disrupt TJ barriers and enhance absorption via the paracellular route. To construct a detection system for interaction between claudin-4 and C-CPE, we prepared purified His (polyhistidine)-tagged claudin-4 (His-claudin-4) and GST (glutathione-S-transferase)-tagged C-CPE (GST-C-CPE) (Supplementary Figure 1) and performed an ELISA to confirm the interaction between His-claudin-4 and GST-C-CPE. By using a range of concentrations of GST-C-CPE, a dose-dependent signal was observed; in contrast, the negative control GST-C-CPE Y306A/L315A showed negligible affinity for claudin-4^19^ (Supplementary Figure 2), indicating that His-claudin-4 interacted with GST-C-CPE.

**Figure 1:**
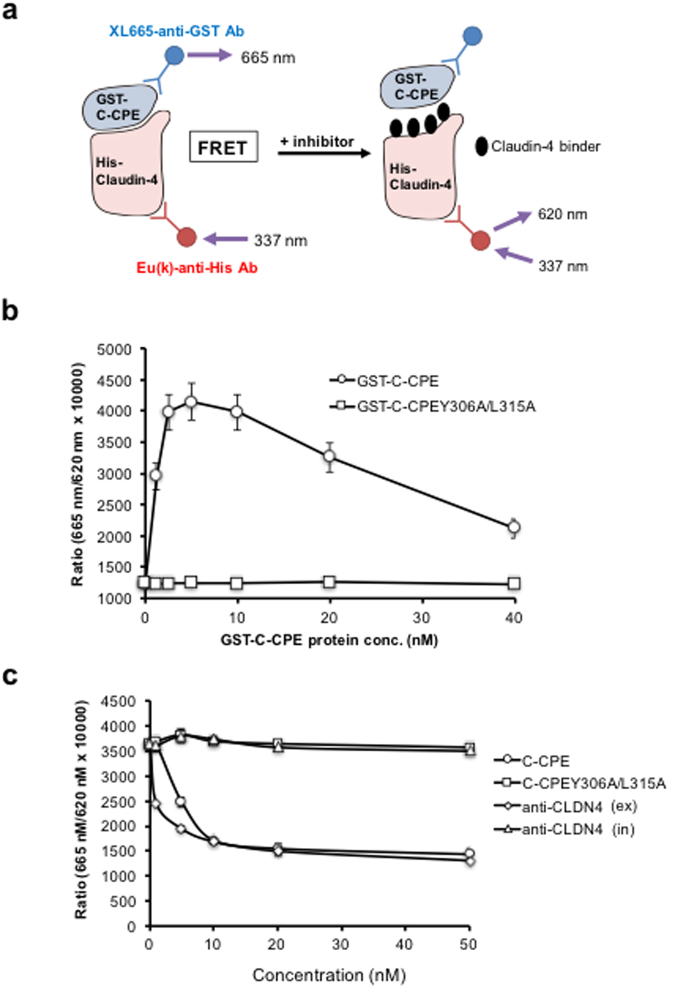
TR-FRET assay for detecting interaction between claudin-4 and C-CPE. (**a**) Schematic representation of the TR-FRET-based assay for detecting the interaction between claudin-4 and C-CPE. When claudin-4 binder binds to the site of claudin-4 and C-CPE interaction, FRET signal is not detected. (**b**) Validation of the TR-FRET-based claudin-4 and C-CPE binding assay. The assay was performed by sequentially adding europium (K)-anti-His antibody (Ab), His-tagged claudin-4 (His-claudin-4) and XL665-anti-GST Ab, followed by the addition of GST-tagged C-CPE or C-CPE Y306A/L315A. TR-FRET signals were measured with an Artemis HTRF plate reader at 620 and 665 nm emission wavelengths. Data are means ± SD (n = 3). (**c**) Inhibitory effect of claudin-4 binders on the interaction between His-claudin-4 and GST-C-CPE in the TR-FRET-based assay. C-CPE, C-CPE Y306A/L315A or anti-claudin-4 Ab (either against the extracellular domain [ex] or the intracellular domain [in] of claudin 4) was incubated with His-claudin-4, and then GST-C-CPE, XL665-anti GST Ab and Eu(K)-anti His Ab were sequentially added. Data are means ± SD (n = 3).

We then developed a novel TR-FRET-based binding assay to detect interaction between claudin-4 and C-CPE. In this assay, europium (K)-conjugated-anti-His- antibody (Eu(K)-anti-His Ab), which binds to His-claudin-4, is used as a donor, and XL-665-conjugated-anti-GST–antibody (XL-665-anti-GST Ab), which binds to GST-C-CPE, is used as an acceptor. The formation of a quaternary complex containing Eu(K)-anti-His Ab, XL-665-anti-GST Ab, His-claudin-4, and GST-C-CPE results in fluorescence energy transfer between donor and acceptor. A binder inhibiting the interaction between claudin-4 and C-CPE disrupts the energy transfer between the donor and acceptor, thus reducing the FRET signal (Fig. 1a). We observed that FRET signals increased after the addition of GST-C-CPE but not after the addition of the negative control C-CPE Y306A/L315A (Fig. 1b). Therefore, the assay successfully detected the interaction of His-claudin-4 with GST-C-CPE. To assess whether the presence of another claudin-4 binder attenuated the FRET signal, we performed competition assays using C-CPEs and claudin-4 antibodies that bind to the C-terminal of claudin-4 (intracellular region) or extracellular domain of claudin-4 (extracellular region)^12^, respectively. The antibody recognising the extracellular domain of claudin-4 reduced TJ-barrier function^12^. Adding C-CPE or the antibody recognising the extracellular domain of claudin-4 considerably reduced the FRET signal, whereas adding C-CPE Y306A/L315A or the antibody recognising the C-terminal of claudin-4 did not affect the signals (Fig. 1c), indicating that this assay can be used to identify binders that bind to the extracellular domain of claudin-4.

### TR-FRET–based screening assay for identification of claudin-4 binders

We optimised the TR-FRET assay for high-throughput screening by focusing on the concentrations of DMSO (dimethylsulfoxide), His-claudin-4, and GST-C-CPE and on the order of addition of compounds in the screening steps; changes in the DMSO concentration (0.1% to 2%) did not alter the FRET signals. The concentrations of C-CPE (2.5 to 10 nM) and claudin-4 (2.5 to 10 nM) that, when combined, yielded the highest FRET signal were each 10 nM. The number of procedural steps and final reaction time that achieved the optimal signal-to-background ratio and Z′-factor value were a 4-step assay and 30 min (Supplementary Figure 3).

**Figure 2:**
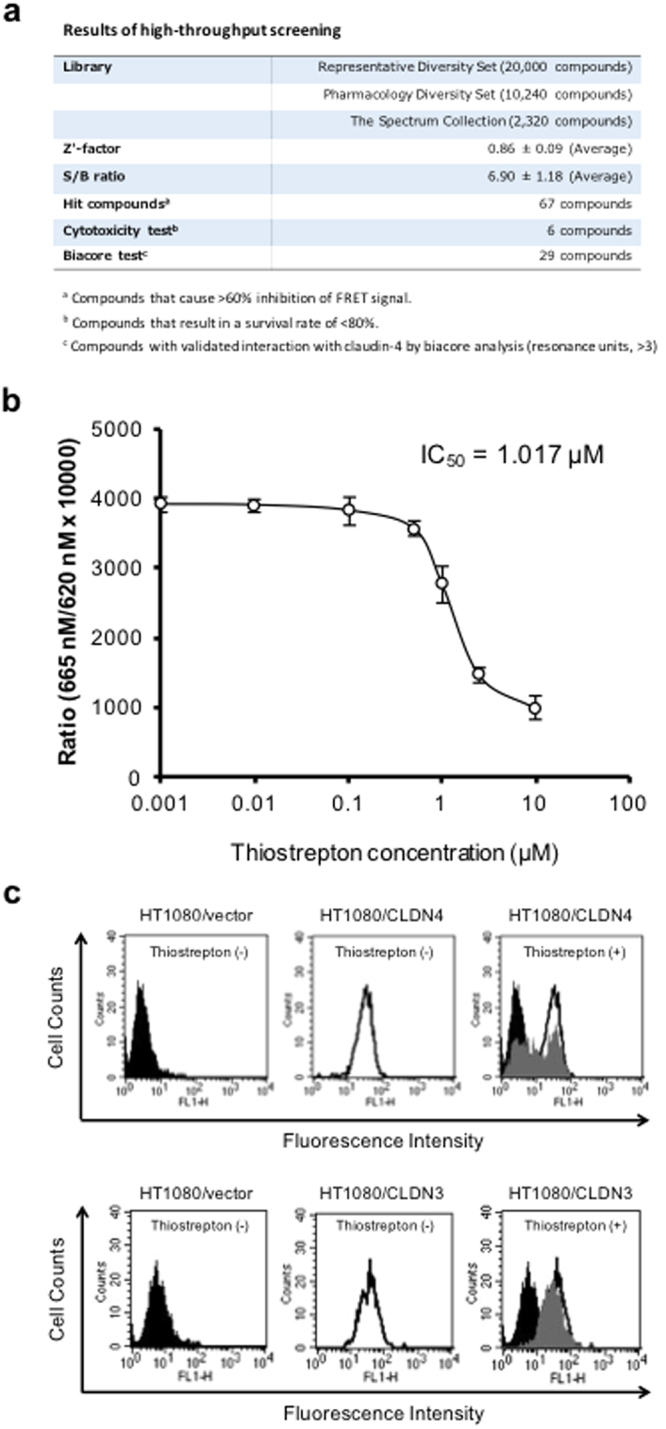
Identification of thiostrepton as a claudin-4 binder. (**a**) Results of the TR-FRET-based high-throughput screening for claudin-4 binders. (**b**) Inhibition of the interaction between claudin-4 and C-CPE by thiostrepton in the TR-FRET assay. Thiostrepton was assayed in triplicate at various concentrations (1 nM to 10 μM) to assess its inhibitory effect. Data are means ± SD (n = 3). IC_50_, concentration at which the inhibitory response was reduced to 50%. (**c**) Competition assay of the interaction of thiostrepton with claudin-3 and claudin-4. Claudin-3- or claudin-4-expressing HT1080 cells (HT1080/CLDN3 or HT1080/CLDN4) and control (claudin non-expressing) HT1080 cells (HT1080/vector) were treated with vehicle alone, 10 µg/mL BSA or 10 µg/mL BSA plus 1 mM thiostrepton for 1 h. Then, FITC-conjugated C-CPE was added to the cells at 20 µg/mL for 1 h. The thiostrepton-bound cells were used for fluorescence-activated cell sorting analysis. Results for cells treated with vehicle alone are displayed in black. Results for cells treated with BSA with or without thiostrepton are displayed in grey or white, respectively.

We used the optimised protocol to screen a 32,560-compound library including (a) the Representative Diversity Set (20,000 compounds), (b) the Pharmacology Diversity set (10,240 compounds), and (c) the Spectrum Collection (2,320 compounds). The assay yielded excellent statistics: the mean Z′-factor was 0.86 ± 0.09, and the mean signal-to-background ratio was 6.9 ± 1.18 (Fig. 2a). The ‘hit’ cutoff was set at >60% inhibition of FRET signal, thus corresponding to 67 compounds. We then conducted a cytotoxicity assay of the hit compounds in human Caco-2 cells and identified 6 compounds with possible cytotoxicity. To determine whether the hit compounds bound directly to claudin-4 protein, not C-CPE, we performed Biacore analysis using recombinant human claudin-4 protein; 29 of the 67 compounds exhibited a Resonance Unit value of >3, thus indicating direct binding to claudin-4. Of the 29 compounds identified in our screen, 4 decreased the transepithelial electrical resistance (TER) value in a Caco-2 cell monolayer (Supplementary Figure 4). These results indicate that the claudin-4 binder screening system based on TR-FRET might be a useful tool for identifying claudin-4 binders with epithelial-barrier-disrupting activity.

Among the hit compounds, thiostrepton achieved the greatest attenuation of the barrier function of Caco-2 cells (Supplementary Figure 4). Thiostrepton inhibited the interaction between His-claudin-4 and GST-C-CPE at an IC_50_ (i.e. the concentration at which interaction is reduced by 50%) of 1.017 µM (Fig. 2b). Biacore analysis of the interaction between claudin-4 and thiostrepton revealed a K_D_ of 6.615 × 10^−6^ (Supplementary Figure 5). In contrast, thiostrepton did not bind to C-CPE. To investigate whether thiostrepton prevents C-CPE from binding to claudins on the cell membrane, we conducted a competition assay. Thiostrepton inhibited the binding of C-CPE to claudin-4 on the cell membrane (Fig. 2c). Thiostrepton also inhibited the interaction between C-CPE and claudin-3, but this effect was substantially weaker than that for claudin-4. These results suggest that thiostrepton preferentially binds to claudin-4 on the cell membrane.

### Thiostrepton attenuates the barrier function of the intestinal epithelial cell monolayer

**Figure 3:**
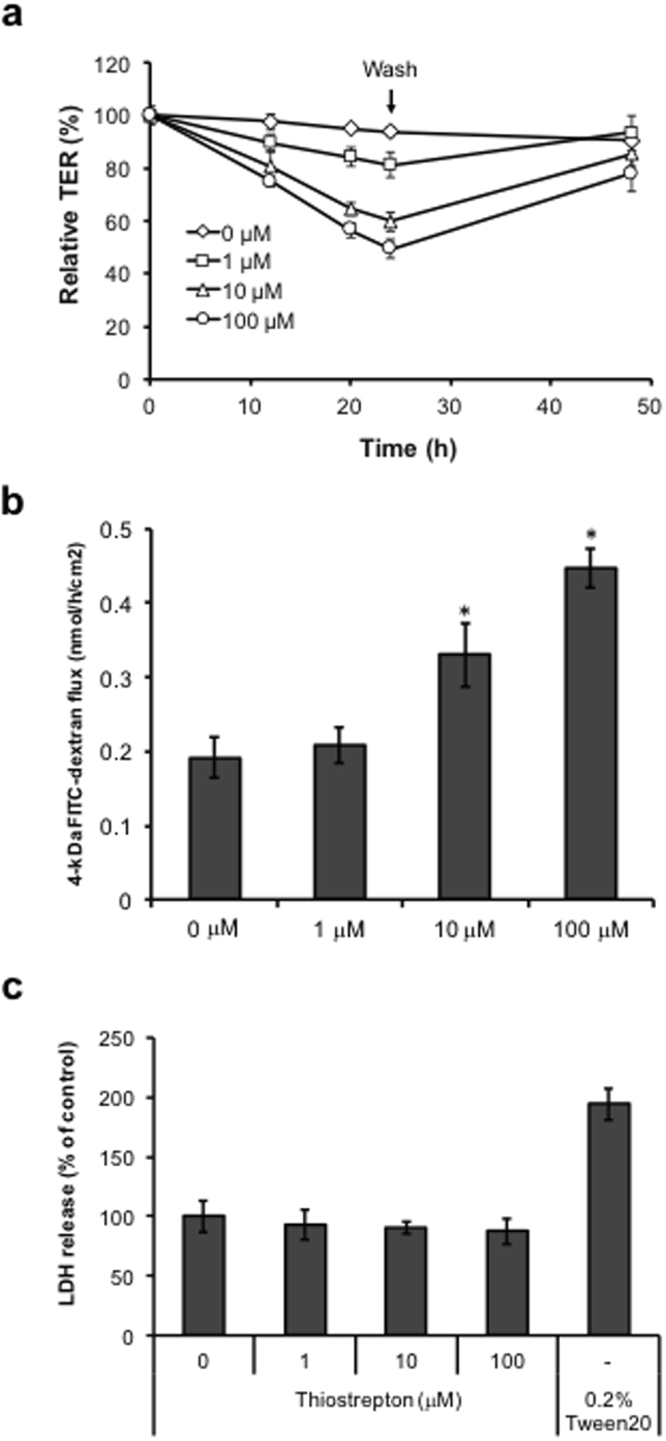
Effect of thiostrepton on the epithelial barrier in a Caco-2 cell monolayer. Caco-2 cell monolayers were incubated with vehicle or 1, 10, or 100 µM thiostrepton. (**a**) After 24 h, the media were replaced with fresh medium, and the cells were cultured for an additional 24 h. TER values were monitored at 12, 18, 24 and 48 h; TER values are shown as a percentage of the TER value at 0 h. Data are means ± SD (n = 3). (**b**) The permeability for 4-kDa FITC–dextran was measured at 24 h. Data are means ± SD (n = 3). *P < 0.05 vs vehicle-treated group, as determined by using Dunnett’s test. (**c**) The viability of cells treated with thiostrepton for 24 h was assessed by measuring the level of lactate dehydrogenase (LDH) released into the culture medium. Tween 20 (0.2%) served as the positive control. LDH release is shown as a percentage of the amount released by the vehicle-treated cells. Data are means ± SD (n = 3).

Thiostrepton is an antibiotic derived from several strains of streptomycetes^20^, but its influence on the TJ barrier is unclear. Therefore, to examine the effect of thiostrepton on the barrier function of epithelial cell monolayers, we treated Caco-2 cells with thiostrepton. Thiostrepton (1–100 µM) reversibly decreased the TER value of Caco-2 cells in a time- and dose-dependent manner (Fig. 3a). We next investigated the effect of thiostrepton on the paracellular permeability of Caco-2 cells by using a paracellular tracer flux assay, with 4-kDa fluorescein isothiocyanate (FITC)–labelled dextran (FD-4) as the tracer. Caco-2 cells were treated with thiostrepton for 24 h, after which we measured the amount of FD-4 that had passed from the apical side to the basal side. Consistent with the TER decrease, thiostrepton (1–100 µM) treatment induced a dose-dependent increase in the paracellular flux of FD-4 (Fig. 3b). Furthermore, thiostrepton was not cytotoxic to Caco-2 cells in the concentration range of 1–100 µM (Fig. 3c). These results indicate that thiostrepton attenuated the TJ-barrier function of Caco-2 cell monolayers.

### Effect of thiostrepton on TJ proteins

**Figure 4:**
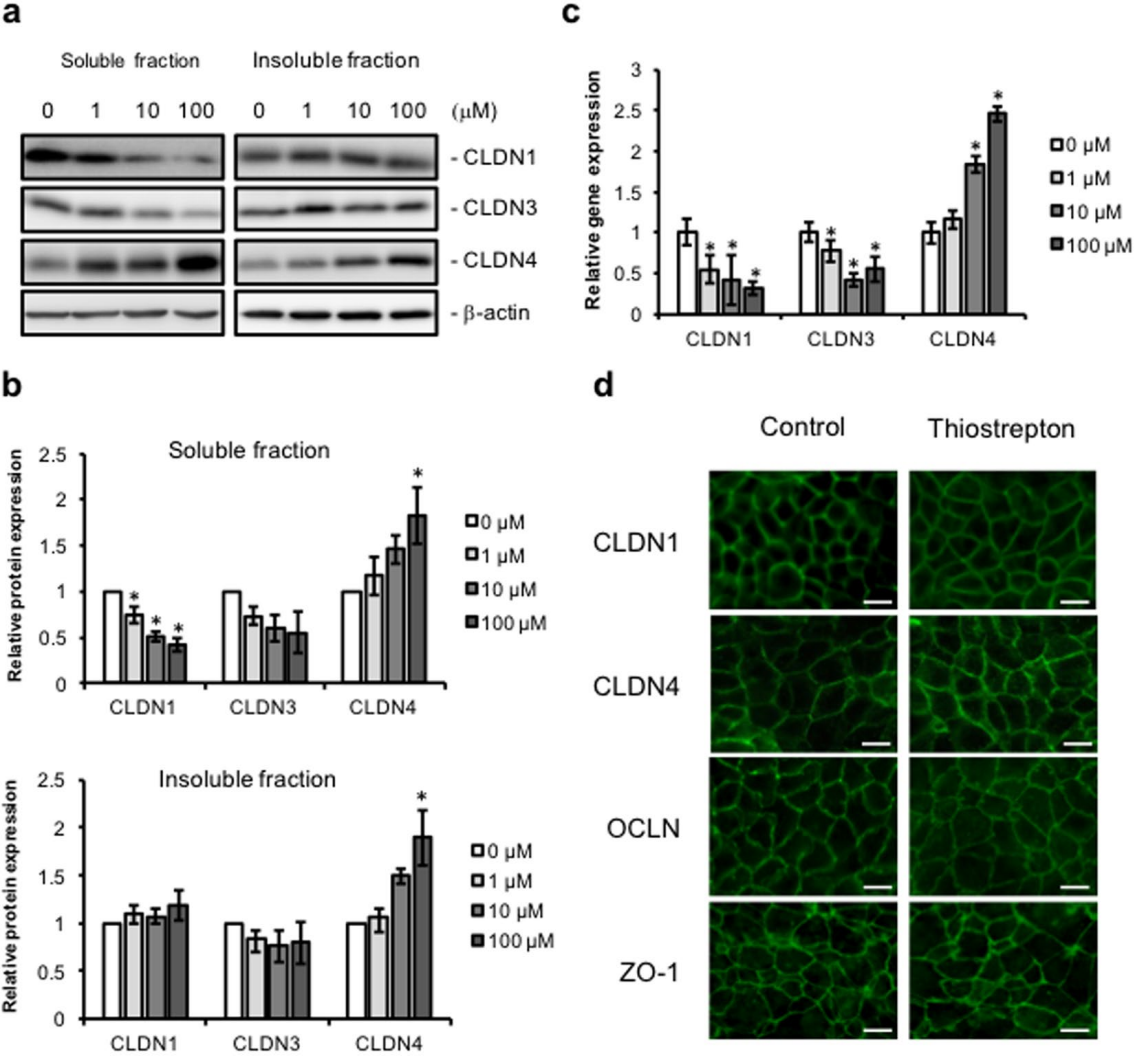
Thiostrepton effect on tight junction components of Caco-2 cell monolayers. Caco-2 cell monolayers were incubated with vehicle or 1, 10 or 100 µM thiostrepton. Triton-X (1%)-soluble and -insoluble lysates were collected and immunoblotted for claudin-1 (CLDN1), claudin-3 (CLDN3) or claudin-4 (CLDN4) (**a**). β-actin served as the loading control. Pre-cropped blots are included in Supplementary Figure 6. Relative protein density was calculated as the ratio of the protein density to the density in the vehicle control (**b**). Data are means ± SD (n = 3). *P < 0.05 vs. vehicle-treated group, as determined by using Dunnett’s test. (**c**) Caco-2 cells were treated with or without 100 µM thiostrepton for 24 h, and then cell lysates were subjected to qRT-PCR analysis for CLDN1, CLDN3, or CLDN4. Data are means ± SD (n = 3). *P < 0.05 vs vehicle-treated group, as determined by using Dunnett’s test. (**d**) Immunofluorescence localisation of CLDN1, CLDN4, occludin (OCLN) and ZO-1 after treatment with 100 µM thiostrepton or no treatment for 24 h. Bar = 10 µm.

**Figure 5:**
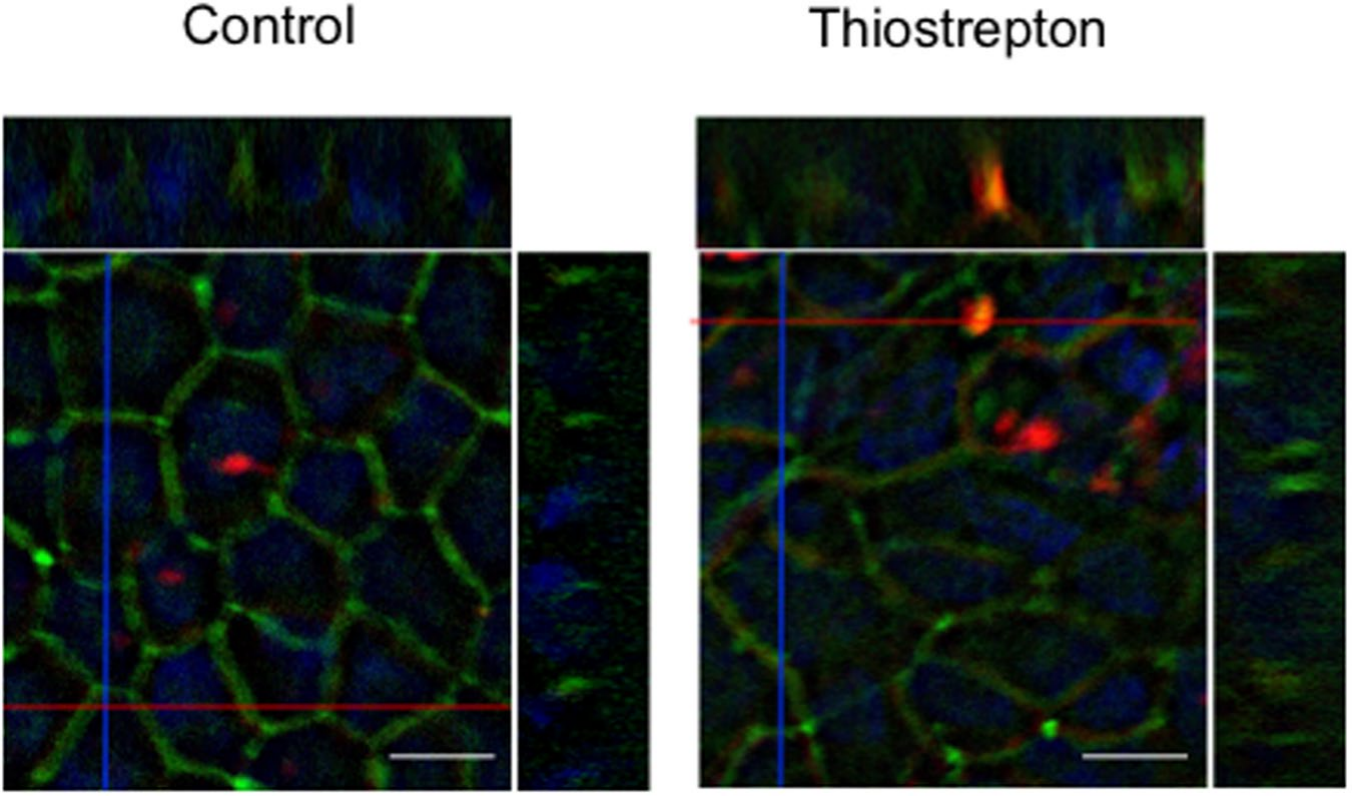
Thiostrepton effect on paracellular tracer permeability. Caco-2 cell monolayers were treated with 100 µM thiostrepton or left untreated (control) and then apically incubated with sulfo-NHS-SS-biotin (606.7 Da; red) and counterstained with anti-ZO-1 antibody (green; TJ stain) and DAPI (blue; nuclear stain). Scanning of the lateral (z-) axis of the cells showed specific localisation of the biotin signals (red) within intercellular contacts, whereas no biotin permeation was observed in control cells. Bar = 10 µm.

To investigate the effect of thiostrepton on TJ components of Caco-2 cells, we separated the 1% Triton-X-soluble and -insoluble fractions and performed immunoblotting and qRT-PCR analyses of cell–cell adhesion molecules. Thiostrepton treatment significantly decreased the 1% Triton-X-soluble protein and mRNA levels of claudin-1 and claudin-3 (Fig. 4a–c and Supplementary Figure 6), whereas the protein levels in the 1% Triton-X-insoluble fraction were unchanged. In contrast, thiostrepton treatment increased the 1% Triton-X-soluble protein and mRNA levels, and the 1% Triton-X-insoluble protein levels, of claudin-4. Immunofluorescence staining showed that thiostrepton treatment did not alter the cellular localisation of claudin-1, claudin-4, occludin or ZO-1 (Fig. 4d). To evaluate the effect of thiostrepton on the paracellular permeation of molecules, we used sulfo-NHS-SS-biotin (607 Da), which is a cell-membrane-impermeable molecule^21,22^. When Caco-2 cell monolayers were treated with thiostrepton and then apically incubated with the biotin compound, biotin signal was detected within the paracellular space; in contrast, no biotin permeation was observed in untreated control cultures (Fig. 5). The signal in the control cultures was due to residual biotin that remained on the apical side after washing; it was not translocated biotin. These results suggest that thiostrepton attenuates TJ-barrier function without distinct changes in the localisation of the TJ components.

### Thiostrepton enhances intestinal absorption of a macromolecule

**Figure 6:**
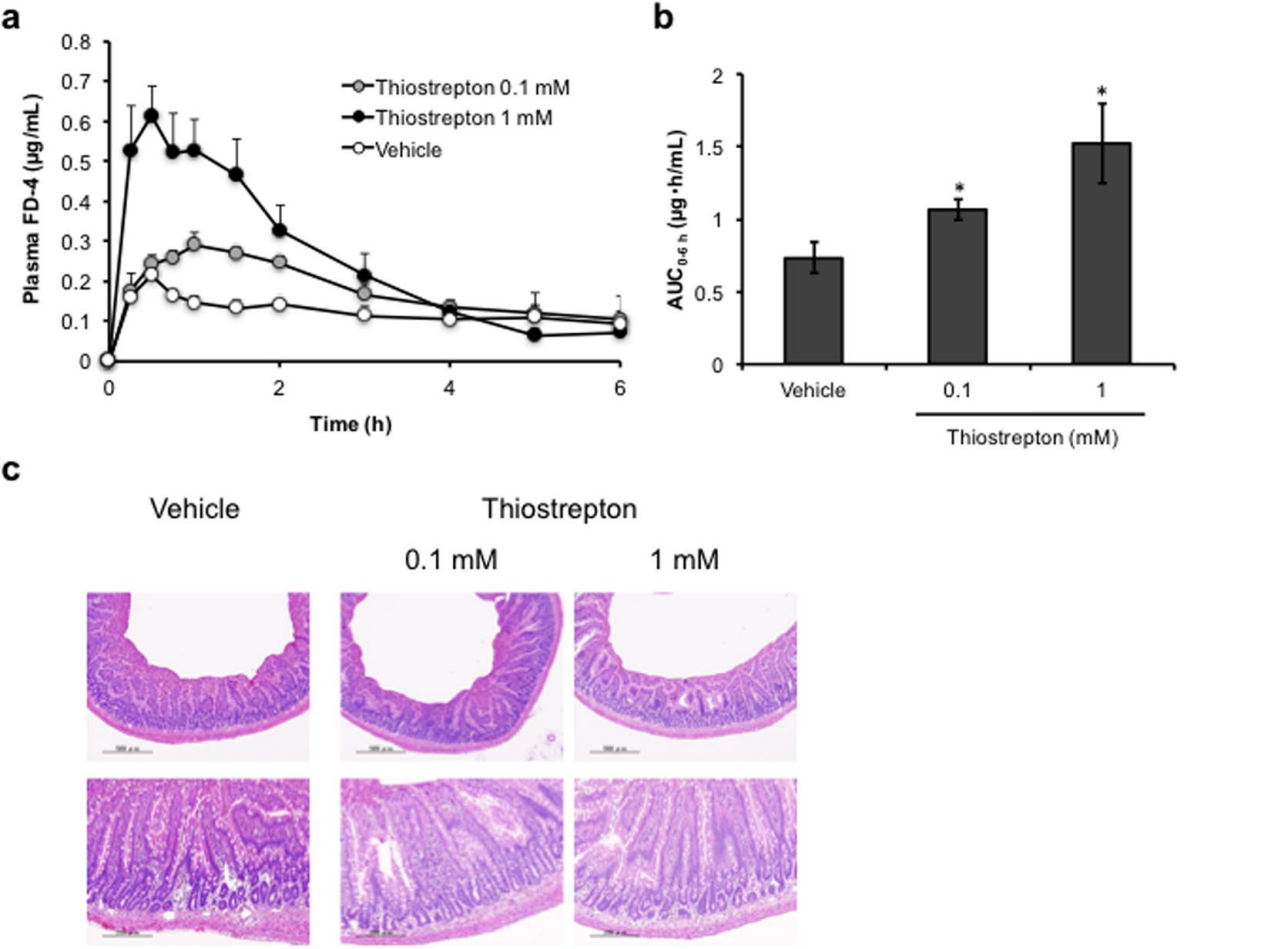
Mucosal absorption-enhancing effect of thiostrepton. (**a**) Rat jejunum was treated with vehicle alone or with 0.1 or 1 mM thiostrepton and 1 mg 4-kDa FITC-labelled dextran (FD-4). Time-course changes in plasma FD-4 concentration were monitored, and the area under the time–concentration curve between 0 and 6 h (AUC_0–6 h_) was calculated (**b**). Data are means ± SD (n = 4). *P < 0.05 vs vehicle-treated group, as determined by using Dunnett’s test. (**c**) Histological analysis of representative thiostrepton-treated rat jejunum. After 6 h of treatment with vehicle or thiostrepton (0.1 or 1 mM), the jejunum was fixed and stained with haematoxylin and eosin.

To investigate the effect of thiostrepton on the jejunal absorption of a macromolecule, we conducted an *in situ* loop assay, using FD-4 as the macro-tracer molecule. Thiostrepton enhanced rat jejunal absorption of FD-4 in a dose-dependent manner, as compared with that in the vehicle-treated group (Fig. 6a and b). In addition, thiostrepton treatment increased plasma FD-4 concentrations, which reached a maximum of 0.6 µg/mL. Histochemical analysis showed no evidence of intestinal tissue toxicity (Fig. 6c and Supplementary Figure 7). These results indicate that thiostrepton has the potential to enhance the intestinal absorption of macromolecules without intestinal toxicity.

## Discussion

Although several claudin-4 binders have been reported^23–25^, C-CPE and its variants are the most commonly reported claudin-4 binders known to regulate TJ barrier function^8,26–28^. C-CPE has the potential to enhance the absorption of macromolecule-like peptides in various mucosal tissues^16,29^. Therefore, modulation of claudin-4-based TJ-barrier function is a promising strategy for developing an absorption enhancer. However, a screening system for identifying claudin binders that might regulate TJ-barrier function had not been developed previously. Here, we constructed a claudin-4 binder screening system based on TR-FRET, which detects the interaction between claudin-4 and C-CPE. We surmise that our novel screening system will not only simplify the identification of compounds binding to claudin-4 but will also restrict the outcome to compounds with TJ barrier-regulating activity, given that the compounds obtained bind substantially to the claudin-4 region that interacts with C-CPE. In fact, we showed that TR-FRET analysis distinguished between an antibody that recognises the intracellular domain of claudin-4, which cannot regulate TJ barriers, and one that recognises the extracellular domain of claudin-4, which attenuates TJ-barrier function. By using this system, we successfully identified novel claudin-4 binders that attenuated epithelial barrier function. Among them, thiostrepton strongly attenuated the TJ-barrier function of Caco-2 cell monolayers. Furthermore, we demonstrated that thiostrepton enhanced rat jejunal absorption of a macromolecule without tissue toxicity. Therefore, our screening system is appropriate for identifying TJ-modulating claudin-4 binders.

Thiostrepton is a veterinary antibiotic used to treat dermatological diseases and mastitis caused by Gram-negative bacteria^20,30^. In addition, thiostrepton shows anti-tumour activity in various human cancer cell lines^31–35^; in melanoma cells, thiostrepton suppresses cell growth and induces apoptosis^36,37^; and in breast cancer cells, thiostrepton leads to cell-cycle arrest and cell death by down-regulating *FOXM1* gene expression^38^. Here, we demonstrated that thiostrepton can attenuate intestinal TJ-barrier function and increase mucosal absorption of macromolecules, indicating that thiostrepton has absorption-enhancing properties. Biacore analysis revealed that thiostrepton directly bound to claudin-4 with a K_D_ of 6.615 × 10^−6^. Competition analysis showed that thiostrepton bound to claudin-4 in the cellular membrane, indicating that claudin-4 is a target molecule for thiostrepton. Thiostrepton also bound to claudin-3 in the cellular membrane, although the affinity for claudin-3 was considerably lower than for claudin-4. C-CPE binds not only to claudin-4 but also to other claudins, including claudins-3, -6, -7, and -9^39^. These findings suggest that the screening system may not completely eliminate, as hit compounds, molecules that bind to other claudins.

TER and tracer flux assays revealed that thiostrepton increased the paracellular permeability of Caco-2 cell monolayers without inducing cell toxicity. Indeed, thiostrepton treatment induced the passage of visibly labelled biotin via the paracellular route. In contrast, the localisation of TJ components, such as claudin-1, claudin-4, occludin and ZO-1, did not change after thiostrepton treatment. These results suggest that the thiostrepton-induced attenuation of barrier function was not due to disruption of the TJ complex but rather perhaps to the disruption of claudin–claudin interactions formed between adjacent cells. Although thiostrepton reduced the TJ-barrier function of Caco-2 cells, it promoted the transcriptional expression and membrane protein production of claudin-4. One possible explanation for this inconsistency may be a reaction to restore the attenuated claudin-4 function. Alternatively, thiostrepton treatment decreased the transcription and cytoplasmic protein production of claudins-1 and -3, whereas the amounts of these proteins in the membrane fraction, which is involved in TJ-barrier function, were not altered. The differences in claudin production levels might be involved with the interaction of thiostrepton with other claudins. The mechanisms underlying the thiostrepton-induced production of claudins are unclear, and future studies need to investigate the effect of thiostrepton on TJ function.

Thiostrepton represents a novel and effective permeability enhancer in intestinal epithelial cells. Partial modification of the structure of thiostrepton might improve its binding properties and specificity. The recent reporting of the structure of the claudin-4–C-CPE complex^40^ will probably facilitate such studies^41^. Although claudin-4 plays a major role in mucosal TJ barriers, its production increases in many malignant tumour types^42^, and it is produced at high levels in epithelial tissues covered with mucosal immune tissues^43,44^. Several reports have indicated that claudin-4 binders are effective in treating cancer (e.g., pancreatic, ovarian, breast, colorectal and gastric cancer cells)^23,24,45–47^ and can be delivered as a vaccine to mucosal immune tissue^44,48,49^. Therefore, the preparation of an effective claudin-4 binder may help to reveal new pharmacological strategies to improve drug delivery, cancer treatment and mucosal vaccine development.

## Methods

### Reagents, antibodies and cells

n-Dodecyl-β-D-maltoside (DDM) was purchased from Dojindo Laboratories (Kumamoto, Japan). FD-4 was purchased from Sigma-Aldrich (St. Louis, MO, USA). Monoclonal anti-6 histidine antibody conjugated europium^3+^ cryptate (Eu(K)-anti-His Ab) and monoclonal anti-GST antibody labelled with XL665 (XL665-anti-GST Ab) were purchased from CIS Bio International (Gif-sur-Yvette, France). Rabbit anti-claudin-1 polyclonal antibody (pAb), rabbit anti-claudin-3 pAb, mouse anti-claudin-4 monoclonal Ab (mAb), mouse anti-occludin mAb and rabbit anti-ZO-1 pAb were purchased from Invitrogen (Carlsbad, CA, USA). Mouse anti-β-actin mAb was purchased from Sigma-Aldrich. Goat anti-rabbit IgG peroxidase-conjugated antibody and goat anti-mouse IgG peroxidase-conjugated antibody were purchased from Millipore (Bedford, MA, USA). Alexa Fluor 488 goat anti-rabbit IgG and Alexa Fluor 488 goat anti-mouse IgG were purchased from Molecular Probes (Eugene, OR, USA).

The human colorectal adenocarcinoma cell line Caco-2 (HTB-37) was obtained from the American Type Culture Collection (Rockville, MD, USA). Caco-2 cells were cultured in Eagle’s minimum essential medium (Nissui, Japan) supplemented with 10% foetal bovine serum under 5% CO_2_ at 37 °C. The passage number of the cells used for the experiments was between 10 and 20.

### Preparation of GST-C-CPEs and C-CPEs

To prepare glutathione-S-transferase-fused C-CPEs (GST-C-CPEs), plasmids encoding C-CPE or C-CPE Y306A/L315A were cloned into pGEX-4T-1 (GE Healthcare, Munich, Germany). The resultant plasmids were transduced into *Escherichia coli* strain BL21 (DE3), after which the cells were cultured in Terrific Broth medium at 37 °C. Isopropyl-1-thio-β-D-galactoside was then added to the cultures, and the cells were grown for an additional 3 h. The cells were solubilised in STE buffer (10 mM Tris-HCl, 150 mM NaCl, 1 mM ethylenediaminetetraacetic acid pH 8.0) containing 10 μg/mL lysozyme, 5 mM dithiothreitol and 1.5% N-lauroylsarcosine. The lysates were centrifuged, and the supernatants were collected and adjusted to 2% Triton X-100. The supernatants were incubated with glutathione–Sepharose beads (GE Healthcare) for 3 h at 4 °C, the beads were washed with STE buffer, and then the GST-C-CPEs were eluted from the beads with STE buffer containing 25 mM glutathione. For preparation of C-CPEs, C-CPEs were cleaved from the GST-C-CPEs with thrombin (Nacalai Tesque, Kyoto, Japan). The solvents were exchanged with phosphate-buffered saline (PBS) by using a PD-10 column (GE Healthcare).

### Preparation of claudin-4 protein

Recombinant human claudin-4 protein was prepared by an expression system using Sf9 cells and recombinant baculovirus^16^. Briefly, the C-terminal His-tagged claudin-4 cDNA fragment was cloned into pFastBac1 (Invitrogen), and recombinant baculovirus was generated by using the Bac-to-Bac baculovirus expression system (Invitrogen). Sf9 cells were infected with the recombinant baculovirus. After 72 h of the infection, the cells were suspended in PBS supplemented with protease inhibitor cocktail (Nacalai Tesque) and 20 units/mL DNase I. The cells were lysed by addition of DDM (to a final concentration of 2%). The resultant supernatant was applied to a HisTrap HP column (GE Healthcare), and His-claudin-4 was eluted with 500 mM imidazole. The solvent for His-claudin-4 was exchanged to PBS containing 0.2% DDM by using a PD-10 column (GE Healthcare).

### TR-FRET assay

In the TR-FRET assay, the Eu(K)-anti-His Ab, which recognises His-claudin-4, was the donor molecule, and the XL665-anti-GST Ab, which recognises GST-C-CPEs, was the acceptor molecule. When His-claudin-4 and GST-C-CPEs were in close proximity, a transfer of fluorescence resonance energy between the donor and acceptor molecules resulted. The assay was performed in a final volume of 100 µL/well in 96-well plates. Eu(K)-anti-His Ab (0.125 nM), His-claudin-4 (10 nM) and XL665-anti-GST Ab (10 nM) were dissolved in TR-FRET buffer (100 mM KF and 0.1% bovine serum albumin [BSA] in PBS) and added to the 96-well plates. Then, GST-C-CPEs (0–40 nM) were added and mixed. After 30 min of incubation at room temperature, FRET signals were measured with an Artemis HTRF plate reader (Berthold Technologies, Wildbad, Germany).

For high-throughput screening, the TR-FRET assay was modified to a 384-well plate format (Supplementary Figure 3). The screening assay was performed in a final volume of 25 μL/well. His-claudin-4 (10 nM) and library compounds (10 µM) were dissolved in TR-FRET buffer and incubated for 1 h. Then, GST-C-CPE (10 nM), XL665-anti-GST Ab (10 nM) and Eu(K)-anti-His Ab (0.125 nM) were sequentially added after each 30-min incubation. FRET signals were measured with an Artemis HTRF plate reader (Berthold Technologies). Library compounds (Representative Diversity Set [20,000 compounds], Pharmacology Diversity set [10,240 compounds] and the Spectrum Collection [2,320 compounds]) were obtained from the Platform for Drug Discovery (Osaka University, Osaka, Japan); these libraries are also commercially available. Detailed information about the libraries can be accessed at their respective web sites: http://www.namiki-s.co.jp/service/screening/kit_product.html and http://www.msdiscovery.com/spectrum.html.

### Flow cytometry analysis

Claudin-3 or claudin-4–producing HT1080 cells were incubated with thiostrepton (1 mM) for 1 h at 4 °C, washed with 1% BSA in PBS, and then incubated with FITC–labelled C-CPE for 1 h at 4 °C. After being washed with 1% BSA in PBS, the cells were analysed by flow cytometry (FACS Calibur, Becton Dickinson, Franklin Lakes, NJ, USA).

### Barrier assays

Caco-2 cells were allowed to differentiate for 10 to 14 days on Transwell filters (diameter, 6.5 mm; pore size, 0.4 µm; Corning, Corning, MA, USA) before their use in TER and paracellular flux assays. Caco-2 cell monolayers had TER values of 600–800 Ω•cm^2^. TER values were measured by using a Millicell-ERS epithelial volt–ohm meter (Millipore Corporation, Billerica, MA, USA). For the paracellular tracer flux assay, 100 µM dialysed FD-4 was added to the medium in the upper chamber. After 1 h of incubation, the medium was collected from the bottom chamber, and the amount of FD-4 was measured with a TriStar LB 941 microplate reader (Berthold Technologies).

### Lactate dehydrogenase release assay

For analysis of thiostrepton-induced cytotoxicity on Caco-2 cells, a lactate dehydrogenase (LDH) release assay was performed according to the manufacturer’s instructions. After being treated with thiostrepton for 24 h, the level of LDH released into the culture medium was analysed. Treatment with 0.2% Tween-20 was used as a 100% lysis control. Absorbance was measured at a wavelength of 570 nm by using a TriStar LB 941 microplate reader (Berthold Technologies).

### Immunoblotting analysis

Caco-2 cells were cultured on a Transwell filter (diameter, 24 mm; pore size, 0.4 µm; Corning). To prepare Triton-X-soluble and -insoluble cell lysates, cells were lysed with 1% Triton-X buffer (50 mM Tris–HCl [pH 7.4], 1.0% Triton X-100, 5 mM ethylene glycol-bis(2-aminoethylether)-N,N,N′,N′-tetraacetic acid) containing a protease inhibitor cocktail (Sigma-Aldrich). Cell lysates were centrifuged at 15,600 × *g* for 5 min at 4 °C to sediment the high-density, actin-rich fraction. The pellet was re-suspended in cell lysis buffer (10 mM Tris–HCl [pH 7.4], 0.3% sodium dodecyl sulphate [SDS]) containing protease inhibitor cocktail. The cell lysates were separated on an SDS–polyacrylamide gel and electroblotted onto polyvinylidene difluoride membranes. The membranes were incubated successively with antibodies against claudins-1, -3 and -4 and β-actin and then with a horseradish-peroxidase-conjugated anti-rabbit or anti-mouse IgG antibody. The reactive bands were detected with Chemi-Lumi One (Nacalai Tesque), and the signals were visualised with ImageQuant LAS4010 (GE Healthcare). The corresponding immunoblot bands were analysed with ImageJ.

### Quantitative reverse transcription – PCR (qRT-PCR) analysis

Total RNA of Caco-2 cells left untreated or treated with thiostrepton for 24 h was isolated by using Sepasol-RNA reagent (Nacalai Tesque), and the RNA (3 μg) was reverse-transcribed by using oligo (dT) primers and a cDNA synthesis kit (Roche, Mannheim, Germany). Quantitative RT-PCR was performed with SYBR Premix Ex Taq II (Takara Bio, Shiga, Japan) by using an Applied Biosystems StepOne Plus system (Applied Biosystems, Foster City, CA, USA). Relative quantification was performed against a standard curve, and the values were normalised against the input determined for the housekeeping gene *glyceraldehyde 3-phosphate dehydrogenase* (*GAPDH*). PCR primers were as described previously^50^.

### Immunofluorescence staining

Localisation of claudins-1 and -4, occludin and ZO-1 on Caco-2 cells was assessed by immunofluorescence microscopy. Caco-2 cells were seeded on Transwell filters (diameter, 12 mm; pore size, 0.4 µm; Corning). After treatment with thiostrepton (100 µM) or no treatment for 24 h, the Caco-2 cells were fixed with methanol–acetone (1:1 v/v) for 5 min and then permeabilised with 0.2% Triton X-100 in PBS for 10 min. After non-specific binding had been blocked by incubation with 1% BSA in TBS buffer (20 mM Tris-HCl, pH 7.4, 40 mM NaCl) containing 0.05% Tween-20 (T-TBS) for 1 h, the cells were incubated with anti-claudin-1, -claudin-4, -occludin and -ZO-1 antibodies in 1% BSA in T-TBS for 1 h. After incubation of the cells with fluorescent secondary antibodies for 1 h, the immunofluorescence images were observed under an immunofluorescence microscope (Keyence, Tokyo, Japan). For visualisation of biotin passing through the TJ, Caco-2 cells treated with 100 µM thiostrepton or left untreated were apically labelled with sulfo-NHS-SS-biotin (606.7 Da) for 10 min. The cells were then fixed, and anti-ZO-1 antibody and 4,6-diamidino-2-phenylindole dihydrochloride (DAPI) were employed for immunofluorescence staining of the TJ or nucleus, respectively.

### *In situ* loop assay

All procedures involving mice and experimental protocols were approved by the Ethics Committee of Osaka University (permission no. 27-5-2). Rat jejunal absorption of FD-4 was evaluated by using an *in situ* loop assay. After 7-week-old Wister male rats were anesthetised, a midline abdominal incision was made and the jejunum was washed with PBS. A 5-cm-long jejunal loop was prepared and both ends were closed with sutures. A mixture of 1 mg FD-4 and 0.1 or 1 µM thiostrepton in 100 µL was injected into the jejunal loop. Blood was collected at the time points indicated. Plasma levels of FD-4 were measured with a TriStar LB 941 microplate reader (Berthold Technologies). The area under the plasma concentration curve of the FD-4 from 0 to 6 h was calculated by using the trapezoidal method. Recovered intestinal tissues were stained with haematoxylin and eosin and examined for tissue toxicity.

### Statistical analysis

Data are presented as means ± 1 SD. Dunnett’s test was used for statistical analyses. A *P* value of less than 0.05 was considered indicative of statistical significance.

## Electronic supplementary material

Supplementary information
