# Supplementary material for: Identification of claudin-4 binder that attenuates tight junction barrier function by TR-FRET-based screening assay
Source: Sci Rep. 2017 Nov 6;7:14514. doi: 10.1038/s41598-017-15108-y (PMC5674027; doi:10.1038/s41598-017-15108-y)
Supplement: Supplementary file 1 — Supplementary information [file 41598_2017_15108_MOESM1_ESM.pdf]

Identification of claudin-4 binder that attenuates tight junction barrier function by TR-FRET-based screening assay

Akihiro Watari[1]*, Miki Kodaka[1], Koji Matsuhisa[2], Yuta Sakamoto[1], Kota Hisaie[1], Norihito Kawashita[3], Tatsuya Takagi[1,4], Yoshiaki Yamagishi[5], Hidehiko Suzuki[6], Hirofumi Tsujino[1] , Kiyohito Yagi[1] and Masuo Kondoh[1]*

[1]Graduate School of Pharmaceutical Sciences, Osaka University, 1-6 Yamadaoka, Suita, Osaka 565-0871, Japan.
[2]Department of Stress Protein Processing, Institute of Biomedical and Health Sciences, Hiroshima University, Hiroshima, Japan.
[3]Faculty of Science and Engineering, Kindai University 3-4-1 Kowakae, Higashiosaka City, Osaka 577-8502, Japan.
[4]Research Institute for Microbial Diseases, Osaka University, 3-1 Yamadaoka, Suita, Osaka 565-0871 Japan.
[5]Research Institute of Pharmaceutical Sciences, Musashino University, 1-1-20 Shinmachi, Nishi-Tokyo, 202-8585, Japan.
[6]Laboratory of Vaccine Materials and Laboratory of Gut Environmental System, National Institutes of Biomedical Innovation, Health and Nutrition (NIBIOHN), Osaka 567-0085, Japan.

***Corresponding authors:**
Akihiro Watari, PhD, Laboratory of Bio-Functional Molecular Chemistry, Graduate School of Pharmaceutical Sciences, Osaka University, Suita, Osaka 565-0871, Japan; Tel: +81-6-6879-8198; E-mail: akihiro@phs.osaka-u.ac.jp
Masuo Kondoh, PhD, Laboratory of Bio-Functional Molecular Chemistry, Graduate School of Pharmaceutical Sciences, Osaka University, Suita, Osaka 565-0871, Japan; Tel: +81-6-6879-8196; Fax: +81-6-6879-8199; E-mail: masuo@phs.osaka-u.ac.jp

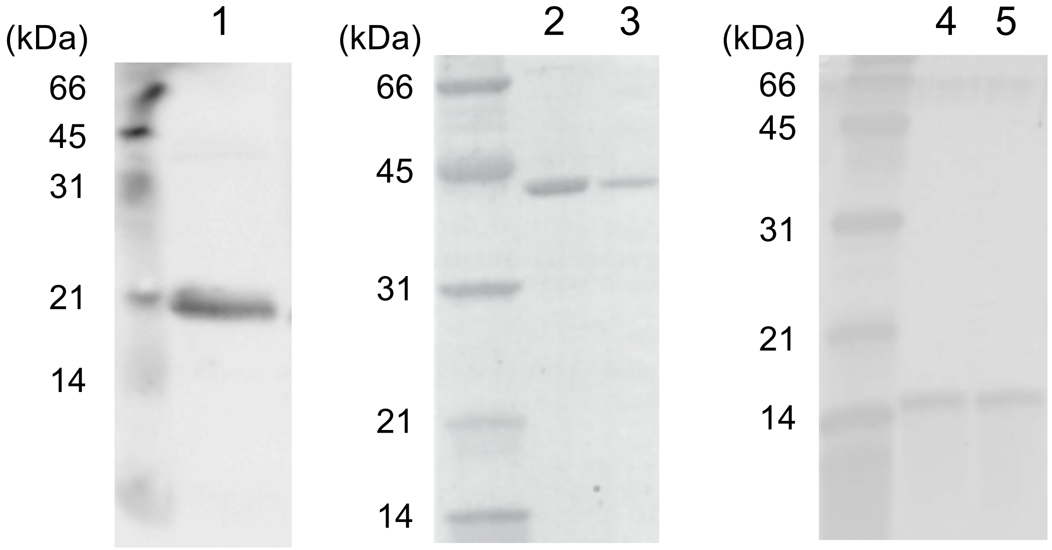

Supplementary Figure 1 Preparation of His-claudin-4, GST-C-CPEs, and C-CPEs. His-claudin-4, GST-C-CPEs and C-CPEs were prepared as described in the Methods. Purification was confirmed by SDS-PAGE (0.5 μg/lane), followed by staining with Coomassie Brilliant Blue. Lane 1, His-claudin-4; lane 2, GST-C-CPE; lane 3, GST-C-CPE Y306A/L315A; lane 4, C-CPE; lane 5, C-CPE Y306A/L315A.

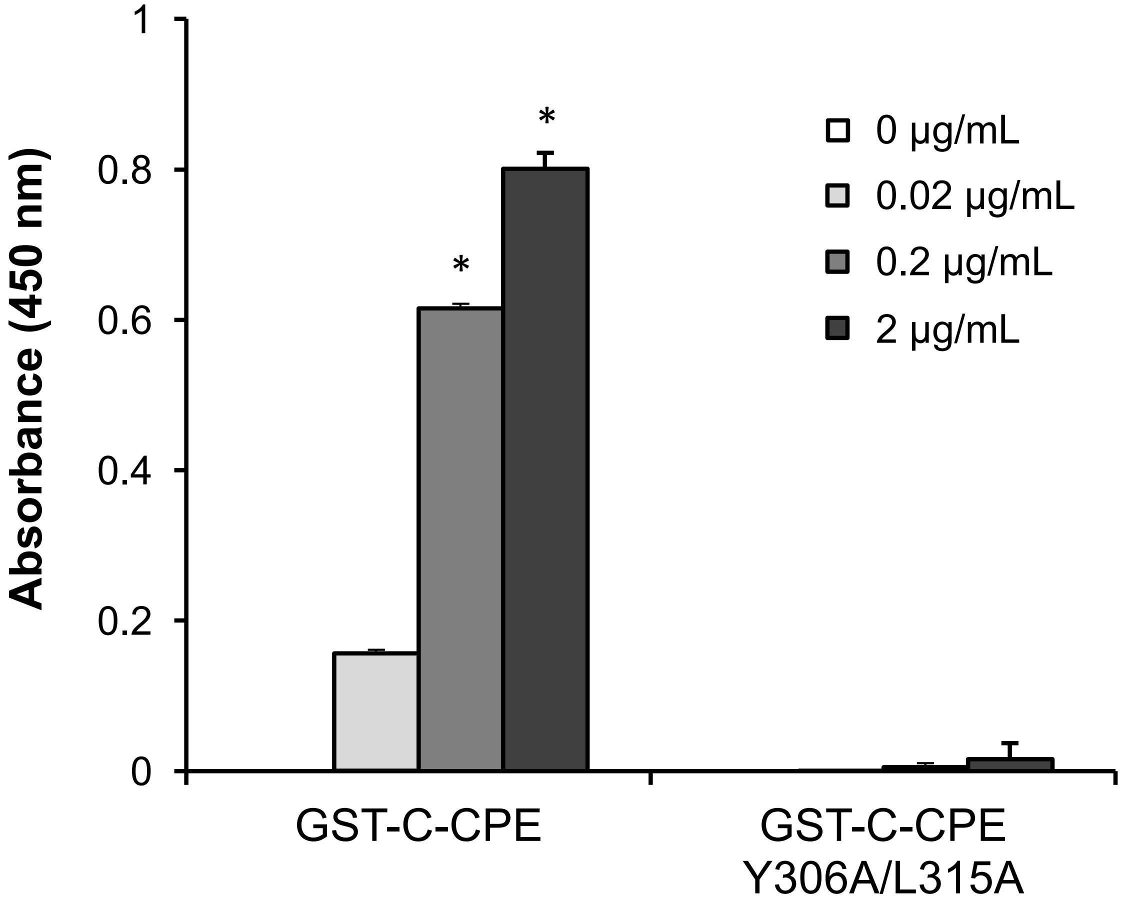

Supplementary Figure 2 ELISA-based binding assay for detection of His-claudin-4 and GST-C-CPE interaction. Plates were coated with His-claudin-4, after which GST-C-CPE or GST-C-CPE Y306A/L315A was added at the indicated concentration. The interactions of His-claudin-4 with GST-C-CPE or GST-C-CPE Y306A/L315A were detected by ELISA analysis. Data are means ± 1 SD (n = 3). *, $P < 0.05$ vs vehicle-treated group, as determined by using Dunnett's test.

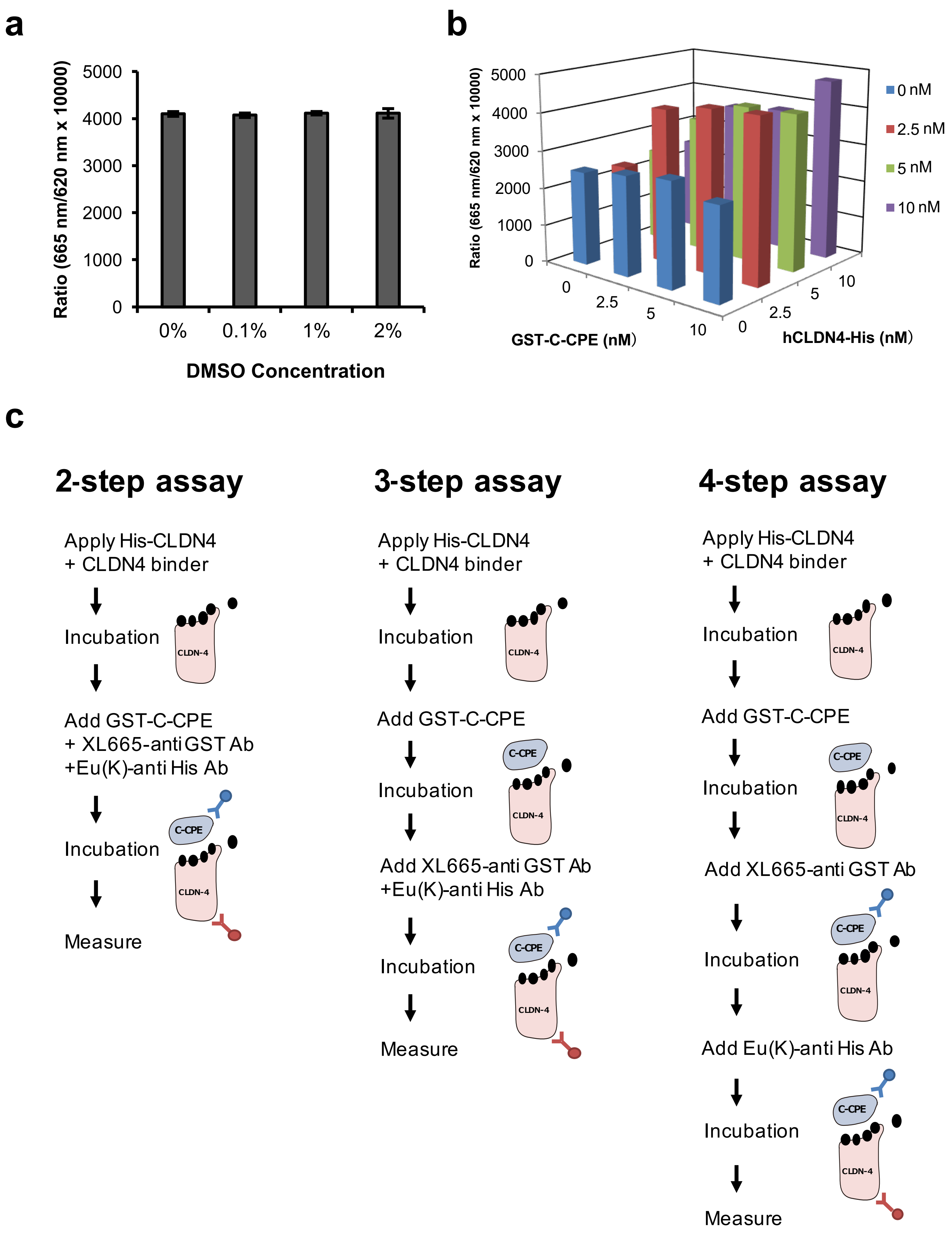

a
Ratio (665 nm/620 nm x 10000)
5000
4000
3000
2000
1000
0
0%
0.1%
1%
2%
DMSO Concentration
b
Ratio (665 nm/620 nm x 10000)
5000
4000
3000
2000
1000
0
0 nM
2.5 nM
5 nM
10 nM
GST-C-CPE (nM)
0
2.5
5
10
hCLDN4-His (nM)
0
2.5
5
10
c
2-step assay
Apply His-CLDN4
+ CLDN4 binder
Incubation
Add GST-C-CPE
+ XL665-anti GST Ab
+Eu(K)-anti His Ab
Incubation
Measure
3-step assay
Apply His-CLDN4
+ CLDN4 binder
Incubation
Add GST-C-CPE
Incubation
Add XL665-anti GST Ab
+Eu(K)-anti His Ab
Incubation
Measure
4-step assay
Apply His-CLDN4
+ CLDN4 binder
Incubation
Add GST-C-CPE
Incubation
Add XL665-anti GST Ab
Incubation
Add Eu(K)-anti His Ab
Incubation
Measure
CLDN-4
C-CPE

d

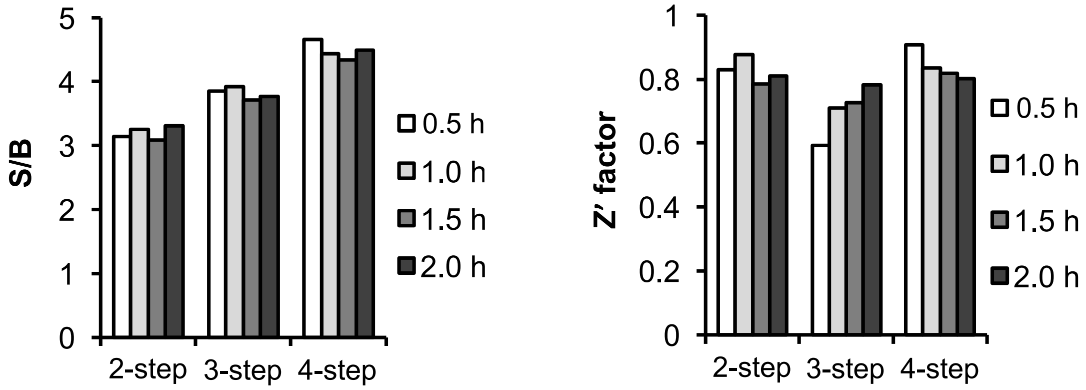

Supplementary Figure 3 Assay optimisation for the claudin-4-C-CPE TR-FRET.
(a) DMSO tolerability of the claudin-4–C-CPE TR-FRET assay. His-claudin-4 (10 nM), GST-C-CPE (10 nM), Eu(K)-anti-His Ab (0.125 nM), XL665-anti-GST Ab (10 nM) and DMSO (final: 0%, 0.1%, 1%, 2%) were dissolved in TR-FRET buffer. After incubation for 30 min, FRET signals were measured. (**b**) Optimal concentrations of His-claudin-4 and GST-C-CPE in the TR-FRET assay. GST-C-CPE (0, 2.5, 5 or 10 nM) and His-claudin-4 (0, 2.5, 5 or 10 nM) with Eu(K)-anti-His Ab (0.125 nM) and XL665-anti-GST Ab (10 nM) were dissolved in TR-FRET buffer. After incubation for 30 min, FRET signals were measured. (**c**) Procedure for each type of assay. In the 2-step assay, His-claudin-4 (shown as pink shapes, with black ovals as claudin-4 binder) was incubated with GST-C-CPE (blue shapes), Eu(K)-anti His Ab (red), and XL665-anti GST Ab (blue). In the 3-step assay, His-claudin-4 was incubated first with GST-C-CPE, after which Eu(K)-anti His Ab and XL665-anti GST Ab were added concurrently. In the 4-step assay, His-claudin-4 was incubated with GST-C-CPE, after which XL665-anti GST Ab and Eu(K)-anti His Ab were added sequentially.
(**d**) For each step of the assay, the mean signal-to-background (S/B) ratio at the indicated final reaction time (0.5, 1, 1.5 or 2 h) was calculated as the average signal ratio divided by the average signal ratio for 0 nM GST-C-CPE. The Z′ factor at the indicated reaction time (0.5, 1, 1.5 or 2 h) was calculated by using the following formula: 1− (3 × SD of each signal ratio + 3 × SD of 0 nM GST-CPE [negative control] signal ratio) / (mean of each signal ratio − mean of negative control signal ratio). TR-FRET signals were measured by using an ARTEMIS plate reader at 620 and 665 nm emission.

Supplementary Figure 4

Compound	Structure	TR-FRET (% of control)[b]	Biacore analysis (RU)[c]	TER (%)[d]
Thiostrepton	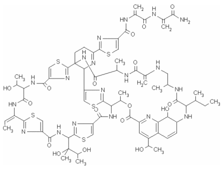	66.24	32.1	55.2
Z20226792[a]	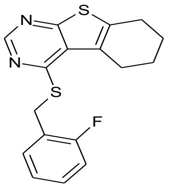	70.07	15.2	72.5
Z55663919[a]	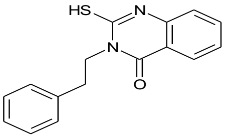	62.43	3.4	70.7
Z32811226[a]	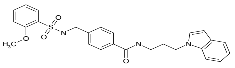	63.75	4.8	90.1

[a] These compounds were purchased from Enamine.

[b] Inhibition of Time-Resolved Fluorescence Resonance Energy Transfer (TR-FRET) signal by interaction between claudin-4 and C-CPE.

[c] Validation of interaction with claudin-4 by biacore analysis. RU, Resonance Units.

[d] The Caco-2 cells monolayers were treated with each compound (10 μM) for 24 h.
Transepithelial electrical resistance (TER) values are shown as percentages of the TER values at 0 h.

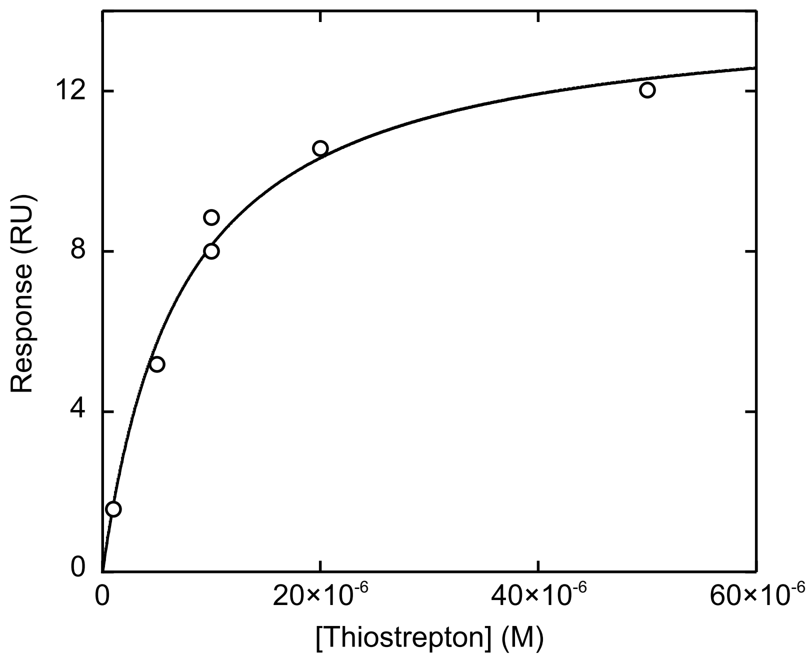

Supplementary Figure 5 Equilibrium analysis of claudin-4 and thiostrepton.
Several concentrations of thiostrepton were plotted against R_{max} values, and a sigmoid curve was fitted to estimate the dissociation constant (K_D).

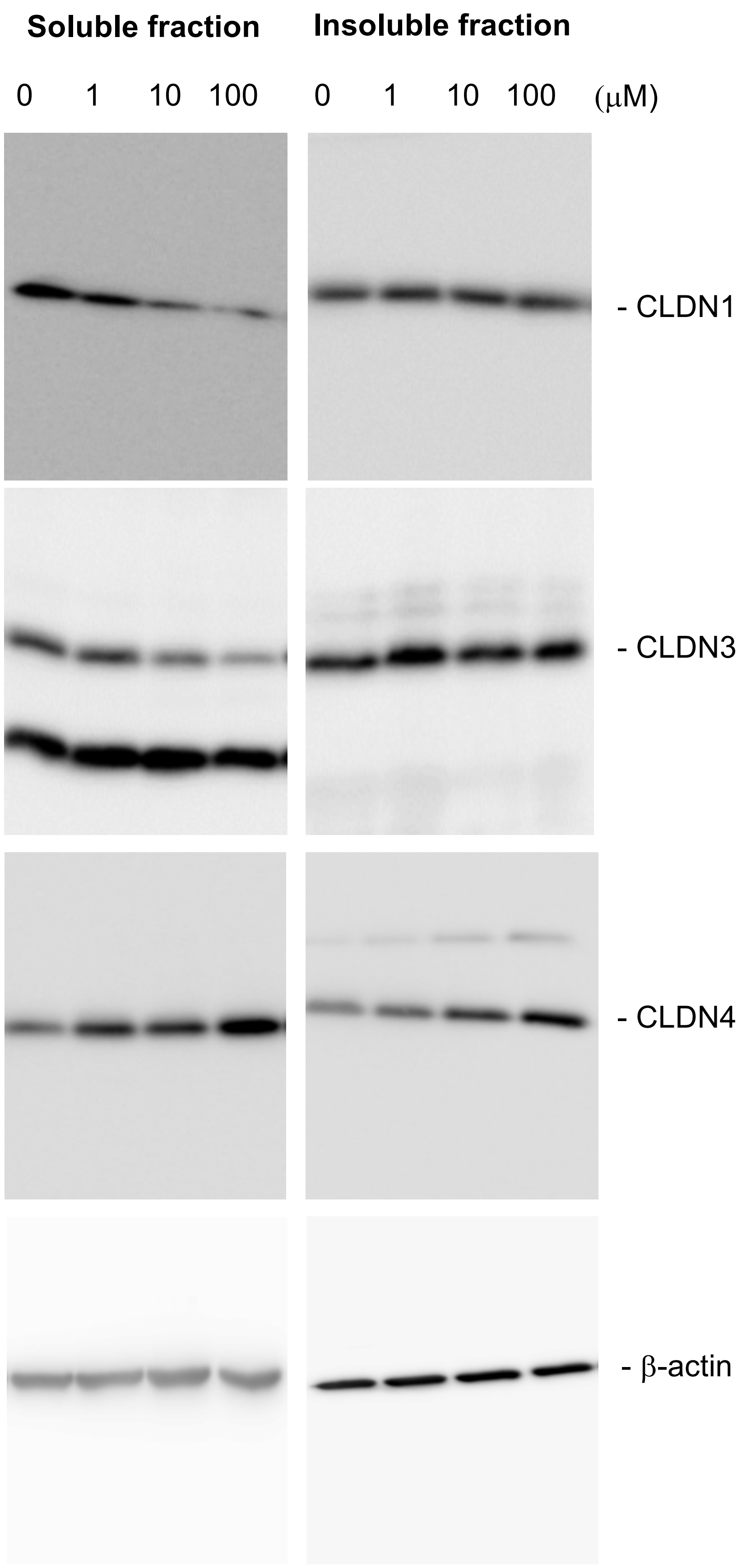

Supplementary Figure 6 Full-length blots for Figure 4a.

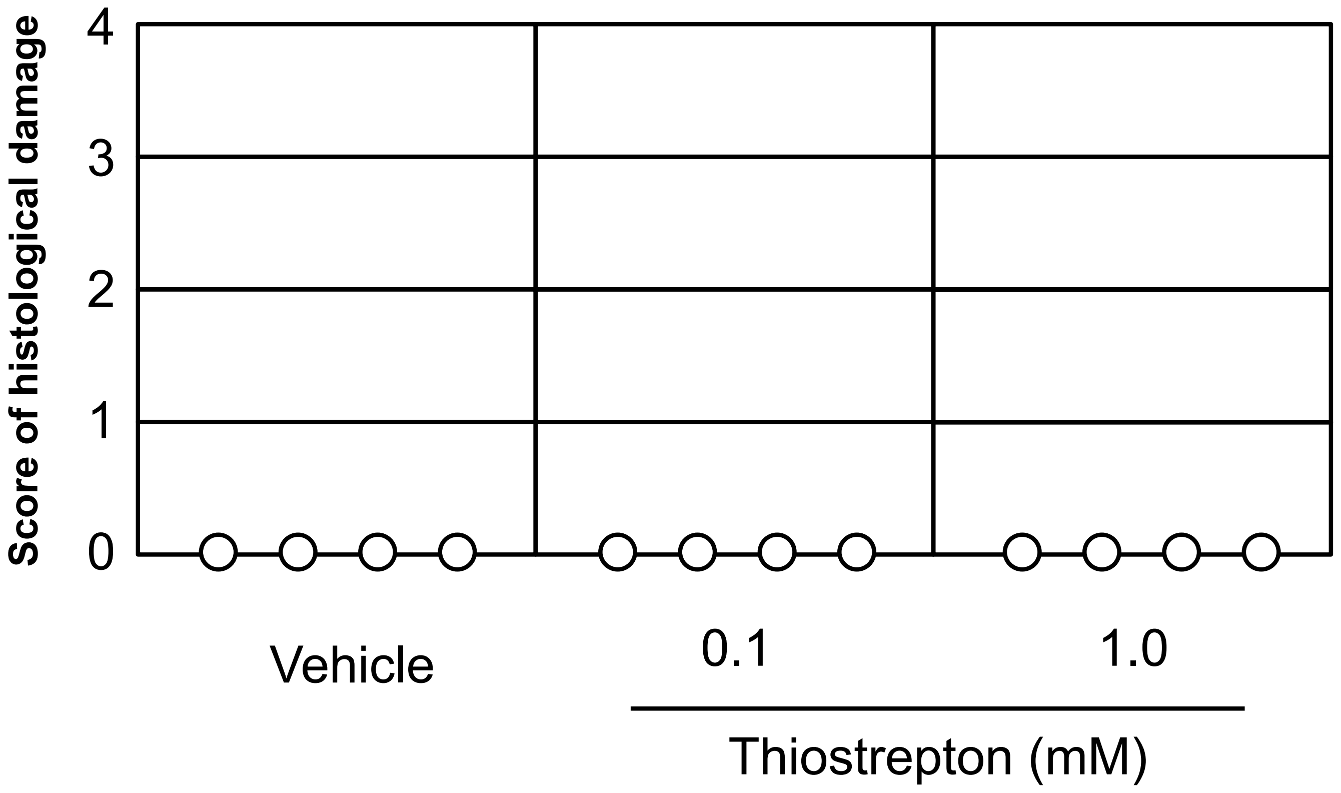

Supplementary Figure 7 Scoring of histological damage of rat jejunum treated with thiostrepton or left untreated. Score 0, normal appearance; Score 1, analysed intestinal tissue showed very slight intraluminal bleeding, erosion, ulceration, stasis, inflammatory cell infiltration, goblet cell reduction, oedema or fibrin-like precipitate; Score 2, analysed intestinal tissue showed slight intraluminal bleeding, erosion, ulceration, stasis, inflammatory cell infiltration, goblet cell reduction, oedema or fibrin-like precipitate; Score 3, analysed intestinal tissue showed moderate intraluminal bleeding, erosion, ulceration, stasis, inflammatory cell infiltration, goblet cell reduction, oedema or fibrin-like precipitate; Score 4, analysed intestinal tissue showed marked intraluminal bleeding, erosion, ulceration, stasis, inflammatory cell infiltration, goblet cell reduction, oedema or fibrin-like precipitate.
